# Identification of a novel, pathogenic *CREBBP* variant in a patient with Menke-Hennekam syndrome: a Case Report

**DOI:** 10.3389/fgene.2025.1585453

**Published:** 2025-08-11

**Authors:** Anna Μaria Anastasiou, Constantia Aristidou, Athina Theodosiou, Ludmila Kousoulidou, Ioannis Papaevripidou, Angelos Alexandrou, Paola Evangelidou, Carolina Sismani, George A. Tanteles, Despina Sanoudou, Aristides G. Eliopoulos

**Affiliations:** ^1^ Department of Biology, School of Medicine, National and Kapodistrian University of Athens, Athens, Greece; ^2^ Clinical Genomics and Pharmacogenomics Unit, 4th Pathology Clinic, School of Medicine, National and Kapodistrian University of Athens, Athens, Greece; ^3^ Department of Clinical Genetics and Genomics, The Cyprus Institute of Neurology and Genetics, Nicosia, Cyprus; ^4^ Department of Cytogenetics and Genomics, The Cyprus Institute of Neurology and Genetics, Nicosia, Cyprus; ^5^ Department of Basic and Clinical Sciences, University of Nicosia Medical School, Nicosia, Cyprus; ^6^ Center of Basic Research, Biomedical Research Foundation of the Academy of Athens, Athens, Greece; ^7^ Genosophy S.A., National and Kapodistrian University of Athens spin-off company, Athens, Greece

**Keywords:** Menke-Hennekam syndrome, CREBBP, genetics, case report, variant, Rubinstein-Taybi syndrome, rare disease

## Abstract

Menke-Hennekam syndrome (MKHK) is a recently described rare autosomal dominant disorder caused by loss-of-function variants in exon 30 or 31 of *CREBBP* (CREB-binding protein) or *EP300* genes. These genes encode transcriptional coactivators with a key role in chromatin remodeling and regulation of gene expression. Herein, we report the identification and characterization of a novel missense variant in *CREBBP*, NM_004380.3:c.5368T>C p.(Cys1790Arg), in a 4-year-old male. The clinical presentation of the patient included global developmental delay, intellectual disability, growth retardation, and distinct craniofacial dysmorphisms, resembling known MKHK subtypes, but also exhibiting less common or unique features such as excessive palmar skin and the absence of recurrent infections and autism spectrum behaviors. Genetic analysis via trio-based clinical exome sequencing confirmed the *de novo* origin of the *CREBBP* variant, which was classified as pathogenic based on ACGS guidelines 2020. Structural modeling predicted that the NP_004371.2:p.(Cys1790Arg) substitution may disrupt the tertiary structure of the CBP TAZ2 domain (amino acids 1772-1840) when interacting with STAT1 but not with adenovirus E1A, potentially affecting transcription factor binding and disease phenotype. The findings contribute to the evolving classification of MKHK subtypes and to deciphering the complexity of genotype-phenotype relationships in MKHK.

## 1 Introduction

Menke-Hennekam (MKHK) syndrome is a rare autosomal dominant disorder caused by heterozygous loss-of-function variants in exons 30 or 31 of the paralogous *CREBBP* and *EP300* genes located on chromosomes 16p13.3 and 22q13.2, respectively. Variants elsewhere in *CREBBP* and *EP300* result in Rubinstein-Taybi syndrome (RSTS1, OMIM # 180849 and RSTS2, OMIM# 613684), which is largely phenotypically distinct. *CREBBP* and *EP300* encode the transcriptional coactivators CREB-binding protein (CBP; OMIM* 600140) and E1A-binding protein 300-KD (p300; OMIM* 602700), respectively, which play critical role in chromatin remodeling, histone acetylation, and transcriptional regulation. CBP and p300 serve as coactivators for several transcription factors, including Notch (Oswald et al., 2001), TP53 (Ferreon et al., 2009a), NFAT (Garcia-Rodriguez and Rao, 1998) and MYC (Faiola et al., 2005), which participate in the regulation of embryonic development, stem cell fate and tissue differentiation (Lipinski et al., 2019; Veneti et al., 2024).

The clinical manifestations of MKHK are heterogeneous and include developmental delay, intellectual disability, craniofacial dysmorphisms, autistic behavior and other systemic abnormalities. MKHK was initially classified into two gene-specific subtypes: MKHK Type 1 (OMIM # 618332), caused by mutations in *CREBBP*, and MKHK Type 2 (OMIM # 618333), caused by mutations in *EP300*. However, recent genotype-phenotype correlations have revised this classification from gene-specific to CBP and p300 domain-specific. In particular, based on variants in TAZ2, ZZ, and ID4 domains, three MKHK subtypes (MKHK-ZZ, MKHK-TAZ2, and MKHK-ID4) have been reported thus far (Haghshenas et al., 2024). TAZ2 is a zinc-finger domain critical for interactions with transcription factors such as TP53 (Ferreon et al., 2009a), STAT1 (Wojciak et al., 2009), E2A (Lochhead et al., 2020), MYC (Ferreon et al., 2009a) and the adenovirus E1A (Ferreon et al., 2009b), the ZZ protein domain mediates binding to acetylated lysines in histones, and ID4 interacts with other chromatin remodeling complexes to regulate transcription. Epigenetic studies have shown that mutations in these domains are associated with distinct CpG methylation patterns, supporting their utility as diagnostic episignatures for MKHK subtypes (Haghshenas et al., 2024).

Herein, we report a novel *CREBBP* missense variant NM_004380.3:c.5368T>C p.(Cys1790Arg) within the TAZ2 domain, identified in a 4-year-old male with MKHK. This case report aims to (1) present the phenotypic features associated with this variant, (2) compare them to domain-specific features reported in the literature, and (3) predict the structural and functional implications of the novel variant towards improving our understanding of MKHK pathophysiology and its clinical implications.

## 2 Materials and methods

### 2.1 Clinical data collection

**FIGURE 1:**
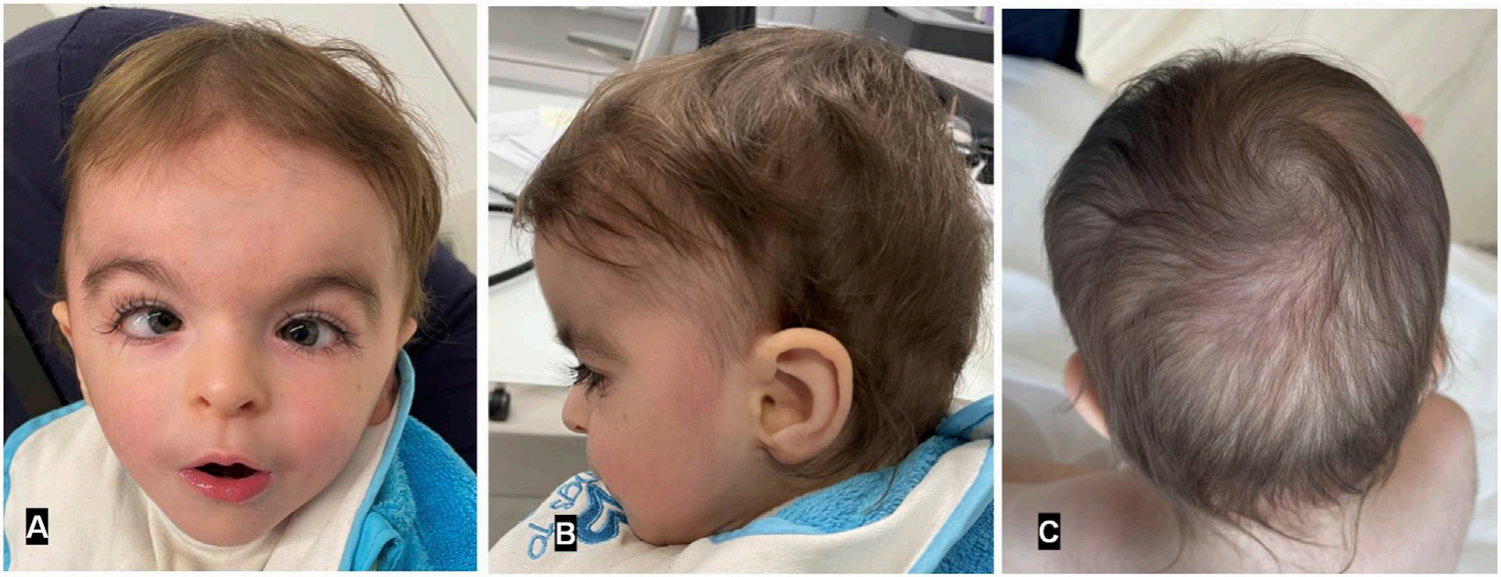
Frontal and profile views of MKHK patient reported herein **(A,B)** at age 2 years and 7 months. Note the broad forehead with hypertelorism, down-slanting palpebral fissures, long eyelashes, squint, increased medial eyebrow separation, tented upper lip, and pointed chin. Faint remnants of a previously reported hemangioma over the occipital region **(C)**.

The patient’s medical history, growth metrics, and developmental milestones were obtained from clinical evaluations conducted at 21, 32, and 40 months. Physical examinations focused on dysmorphic features, systemic anomalies, and developmental delays. Family history was collected to assess inheritance patterns. This case report is a retrospective analysis of clinical genetic testing performed as part of routine diagnostic evaluation. According to institutional policies, formal approval by the Ethics Committees is not required for single-patient case reports without experimental interventions. Written informed consent for publication (including the photographs in Figure 1) was obtained from the patient’s legal guardians.

### 2.2 Genetic analysis

Trio-based Clinical Exome Sequencing (CES) was performed on the Illumina NextSeq 2000 platform using the TruSight One sequencing panel covering ∼4,800 genes selected from OMIM, HGMD, GeneTests, and additional clinically curated sources, encompassing genes with established or emerging associations to human disease. Bioinformatic analysis, annotation and interpretation were performed with VarSome Clinical (Kopanos et al., 2019) (version: 11.3) using the human reference genome build hg19. Variants were classified based on the recommendations from ACGS guidelines 2020 (https://www.acgs.uk.com/quality/best-practice-guidelines/) sequence variant interpretation working group (https://clinicalgenome.org/working-groups/sequence-variant-interpretation) and the automatic scoring ACMG classification of VarSome Clinical (version: 11.3). Sanger sequencing was used to validate any candidate variants identified by CES.

### 2.3 Structural modeling

The structural impact of the identified CREBBP variant was analyzed using Missense3D-DB (https://missense3d.bc.ic.ac.uk/∼missense3d/) (Khanna et al., 2021). The focus was on modeling the putative effects of TAZ2 variants on the structure of CBP-TAZ2 domain in complex with transcription factors, such as E1A and STAT1. The relevant nuclear magnetic resonance (NMR) structures were downloaded from the RCSB Protein Data Bank (PDB, https://www.rcsb.org/) using the terms *CBP*, *TAZ2* and *homo sapiens* (PDB IDs: 2KJE and 2KA6, respectively).

### 2.4 Literature review

A review of MKHK literature was conducted in PubMed using the terms “Menke-Hennekam syndrome AND CREBBP” to identify phenotypic characteristics associated with the *CREBBP* variants, particularly those affecting the TAZ2 domain. Only full-text manuscripts in English published in peer-reviewed journals up to 20 February 2025 were included in the review.

## 3 Results

### 3.1 Clinical presentation

The clinical features of the patient, born to Greek-Cypriot parents, are provided in Table 1. The patient is a 4-year-old male, the first-born twin of a dichorionic diamniotic pregnancy, who was delivered at 36 weeks of gestation by cesarean section due to maternal indications. The pregnancy was complicated by polyhydramnios, but no structural abnormalities were identified prenatally. The patient’s weight at birth was 2.1 kg (<10th centile), consistent with intrauterine growth restriction. Neonatal complications included hypertonia, although no immediate interventions were required.

**TABLE 1:** Patient characteristics.

Age (years)	4 years and 3 months
Gender (M/F)	M
CREBBP mutation	c.5368T>C
Face,square (S)/flat (F)	F
Telecanthi(T)/ Epicanthi (E)	No
Palpebral fissures upslanted (U)/Downslanted(D)	D
Palpebral fissures, short	No
Ptosis	No
Squint	Yes
Depressed Nasal Ridge	Yes
Short nose	Yes
Broad nasal tip	Yes
Short columella	Yes
Anteverted nares	No
Full cheeks	No
Philtrum short (S)/ long (L) / deep (D)	D
Everted vermilion of upper lip	No
Thin vermilion of upper lip	No
High palate	No
Micro/retrognathia	No
Ears low-set (L)/ short (S)	No
Protruding ears (upper part)	No
Cupped ear	No
Overfolded helix	No
Ulnar deviation of finger (s)	No
Clinodactyly fifth finger	No
Prominent fetal tip pads	Yes
Sandal gap	Yes
Cutaneous partial syndactyly of toes	No
Fibular deviation distal phalanx	No
Halluces broad (B)/ narrow (N)	No
Other*	CN, UT
Prenatal growth retardation	Yes
Postnatal growth retardation	Yes
Microcephaly (OFC<3rd centile)	Yes (minus 2.9SD below mean)
Hypertrichosis	Yes (mildly hirsuit back)
Highly arched eyebrows	Yes
Long eyelashes	Yes
Down-slanted palpebral features	Yes
Convex nasal ridge	Yes
Low hanging columella	No
Grimacing Smile	No
Broad thumbs	No
Angulated thumbs	No
Broad halluces	No
Apparent intellectual disability/ developmental delay	Yes, Severe
Epilepsy	No
Autism/Autism-like behaviour	No
Cardiovascular anomalies	No
Urinary tract anomalies	No
Scoliosis	No
Obesity	No

FU, frontal upsweep of hair; LE, long eyelashes; BE, broad eyebrows; DE, deep-set eyes; EI, extra incisor; Cr, cryptorchidism; MC, megalocornea; LC, low hanging columella; CN, convex nasal ridge; DC, dolichocephaly; UT, unerupted teeth; TP, tapering fingers; Camp, camptodactyly, OT, overlapping toes, AH, anteriorly implanted hallux, PT, pointy canine teeth, AE, absent lobe of the ear.

Developmental delays were evident from infancy. Key milestones were severely delayed, including unsupported sitting that was achieved at 24 months, and walking with support achieved at 32 months. Speech development was also severely delayed, with communication limited to nonverbal sounds and gestures. Neurological evaluations noted persistent hypertonia, but no focal neurological deficits were identified. Despite some improvements in motor coordination over time, global developmental delays remained significant.

Anthropometric measurements at 21, 32, and 40 months confirmed growth retardation. At 21 months, the patient’s weight was 7.4 kg (−3.9 SD below the mean for age and gender), height was 74.5 cm (−3.7 SD) and occipitofrontal circumference (OFC) was 43.5 cm (−3.2 SD). By 40 months, these parameters remained below the expected range for age and sex with body weight being 9 kg (−4.3 SD), height at 85 cm (−4.2 SD), and OFC at 45.3 cm (−3 SD). Dysphagia and oromotor incoordination persisted throughout evaluations, causing a restricted diet and poor appetite that contributed to poor weight gain, necessitating a diet of pureed foods.

The patient exhibited distinctive craniofacial anomalies, including sparse hair, long, unruly eyelashes, highly arched eyebrows with minimal synophrys (more evident in infancy), a depressed nasal bridge, a short nose with anteverted nares, a short columella, and a long philtrum (Figure 1). Hypodontia was also noted, with delayed eruption of primary teeth. No abnormalities were observed in the palate or uvula. External genitalia were unremarkable male. Additional systemic anomalies were noted upon physical examination such a hirsute back, hypoplastic fifth toenails and excessive palmar skin. A hemangioma over the occiput was also observed but resolved by 12 months of age. Detailed ophthalmologic and auditory evaluations were inconclusive due to poor cooperation. Middle ear effusion was also detected. Notably, the patient did not display recurrent infections, gastroesophageal reflux or pathological cardiovascular findings, previously reported in some but not all MKHK patients.

Behaviorally, the patient demonstrated a calm temperament with limited engagement in social interactions. While some MKHK patients exhibit autistic traits or self-injurious behaviors, these features were absent in this case. However, the absence of verbal communication and reliance on nonverbal cues significantly hindered social interactions.

Serial assessments revealed gradual improvements in motor skills, though intellectual disability remained profound. Feeding difficulties persisted as a prominent issue, requiring ongoing nutritional interventions. Despite the absence of life-threatening complications, the patient’s overall developmental trajectory necessitated multidisciplinary management, including physical therapy, speech therapy and nutritional support.

The family history was unremarkable for genetic disorders, intellectual disabilities, or congenital anomalies. The patient’s twin sibling exhibited minor anomalies, including metopic synostosis and a right foot deformation, but no significant developmental delays or systemic issues.

### 3.2 Genetic analysis

Previous array comparative genomic hybridization (array-CGH) analysis, using SurePrint ISCA array (Agilent-version 5.1) with 60,000 oligos, gave normal results [arr (X,Y)x1, (1-22)x2].

**FIGURE 2:**
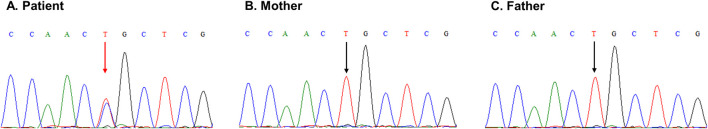
Sanger sequencing electropherograms demonstrating the presence of the *CREBBP* NM_004380.3:c.5368T>C p.(Cys1790Arg) variant at NC_000016.9:3779680 in the patient **(A)** (red arrow), as identified by VarSome Clinical v11.3, and its absence from the mother **(B)** and the father **(C)** (black arrows).

CES identified a heterozygous *de novo* missense c.5368T>C variant in *CREBBP* (NM_004380.3) resulting in a cysteine to arginine substitution at codon position 1790 (Cys1790Arg) within the TAZ2 zinc-finger domain. The identified c.5368T>C variant was classified as pathogenic using the criteria PM2 supporting, PM1 moderate, PP3 strong and PS2 strong. The variant was absent from the Genome Aggregation Database (gnomAD), it is located in a mutational hot spot, *in silico* tools strongly support its pathogenicity and the *de novo* origin of the variant was confirmed by parental genotyping using Sanger sequencing (Figure 2). No additional candidate pathogenic or likely pathogenic variants were identified.

### 3.3 Phenotypic comparison with MKHK subtypes

Although the initial clinical suspicion leaned toward a broader category of chromatinopathies including Cornelia de Lange (CdL) syndrome (Rohatgi et al., 2010), the identification of a pathogenic variant in the TAZ2 domain of CREBBP led to a focused re-evaluation of the patient’s craniofacial and neurodevelopmental profile. This reverse phenotyping confirmed a striking overlap with the Menke-Hennekam syndrome spectrum.

Our literature review identified eight relevant papers. Overall, MKHK subtypes display a variable phenotype (Haghshenas et al., 2024; Banka et al., 2019; Nishi et al., 2022; Sima et al., 2022; Menke et al., 2016; Menke et al., 2018; Cogan et al., 2023; Angius et al., 2019), which is evident even among individuals bearing the same *CREBBP* variant (Haghshenas et al., 2024; Sima et al., 2022). When compared to previously reported MKHK cases, the patient reported herein exhibited a combination of common and unique features. Key characteristics such as intellectual disability, microcephaly, feeding difficulties, and growth retardation aligned with the typical phenotype of MKHK Type 1. However, the absence of certain features, such as autistic or aggressive behaviors, recurrent infections, and specific craniofacial anomalies like a long philtrum or short palpebral fissures, differentiates this patient from the majority of previously reported MKHK cases.

Based on the recent domain-specific classification (Haghshenas et al., 2024), the patient’s phenotype mostly aligns with MKHK-TAZ2. Overlapping features include intellectual disability, feeding problems, and anomalies at the extremities (e.g., hypoplastic toenails). Unique to this case were excessive palmar skin and the absence of gastroesophageal reflux, autism spectrum behavior or recurrent infections that are found in several (but not all) MKHK-TAZ2 patients (Haghshenas et al., 2024; Nishi et al., 2022; Sima et al., 2022) (Supplementary Table 1).

While the patient reported herein did not face cultural or financial barriers, diagnostic challenges in MKHK syndrome may more broadly arise from limited availability of targeted genetic testing and the variability of early clinical features.

### 3.4 Structural modeling of the NP_004371.2:p.(Cys1790Arg) variant

**FIGURE 3:**
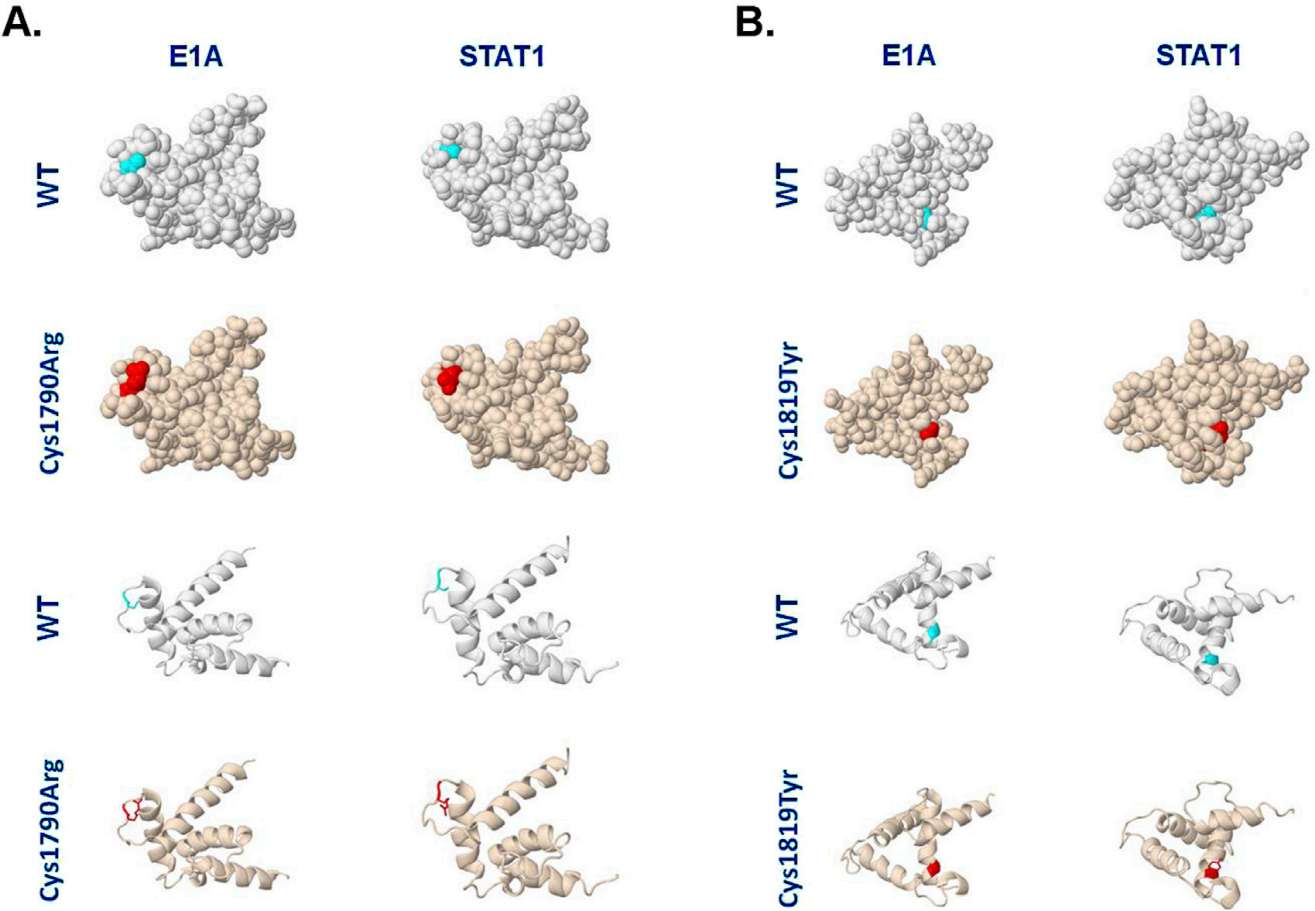
Different TAZ2 variants are predicted to differentially affect the TAZ2 structure when complexed with E1A or STAT1. **(A)** Models of human CBP TAZ2 in complex with E1A or STAT1-interacting regions and the predicted effects of Cys1790Arg substitution. The NMR structures were retrieved from the Protein Data Bank (https://www.rcsb.org/) with IDs 2KJE and 2KA6, respectively, and analyzed on the Missense3D platform (https://missense3d.bc.ic.ac.uk/). The light blue color depicts the position of Cys1790 and the red color depicts the Arg1790 variant. **(B)** Models of human CBP TAZ2 NMR structure in complex with E1A or STAT1-interacting regions, and the predicted effects of Cys1819Tyr substitution. The light blue color depicts the position of Cys1819 and the red color depicts the Tyr1819 variant. Spacefill and ribbon models are shown. See text for details.

Structural modeling of the NP_004371.2:p.(Cys1790Arg) variant was performed using the Missense3D platform (Khanna et al., 2021). This *in silico* analysis predicted that the Cys1790Arg substitution does not affect the tertiary structure of the TAZ2 domain in complex with the adenovirus E1A CR1 region (residues 53–91; Figure 3A) (Ferreon et al., 2009b). Specifically, the 3D model predicts that both the wild-type Cys^1790^ and the mutated Arg residue are similarly exposed, having Relative Solvent Accessibility (RSA) 20.0% and 37.5%, respectively. In contrast, Cys1790Arg was predicted to affect the structure of TAZ2 when it is complexed with the transactivation domain of STAT1 (Wojciak et al., 2009). Specifically, whereas Cys^1790^ is buried in this structure (RSA 3.7%), Arg is exposed (RSA 26.6%).

For comparison, we also analyzed the structural changes predicted to occur as a result of another Cys substitution in TAZ2, Cys1819Tyr, which has been previously linked to MKHK syndrome (Haghshenas et al., 2024). Interestingly, unlike Cys1790Arg (Figure 3A), the Cys1819Tyr substitution does not appear to affect the structure of TAZ2 in complex with STAT1 (Figure 3B). However, the latter substitution was predicted to disrupt all side-chain/side-chain H-bonds and/or side-chain/main-chain H-bonds formed by the buried Cys^1819^ residue (RSA 0.0%) of CBP-TAZ2 when complexed with E1A. Additionally, the Cys1819Tyr substitution is predicted to result in a change between buried and exposed state of the target variant residue with Cys being buried (RSA 0.0%) and Tyr partly exposed (RSA 11.7%).

## 4 Discussion

Pathogenic variants in the TAZ2, ZZ, and ID4 regions of CBP and p300 are central to genetic classification of Menke-Hennekam syndrome (Haghshenas et al., 2024). Herein, we report a novel *CREBBP* missense variant, NM_004380.3:c.5368T>C, which results in the substitution Cys1790Arg within the TAZ2 domain of CBP. While *CREBBP* variants in exons 30–31 have been associated with Rubinstein-Taybi syndrome, the CBP TAZ2 domain spanning amino acid residues 1772–1840 is a functionally distinct region, as variants within TAZ2 are now consistently linked with MKHK and a specific DNA methylation signature (Haghshenas et al., 2024; Nishi et al., 2022; Sima et al., 2022). The absence of Rubinstein-Taybi hallmarks (e.g., broad thumbs, angulated thumbs, characteristic nose/columella) and the presence of MKHK-TAZ2 specific traits (Supplementary Table 1) support the classification of our patient within the MKHK-TAZ2 subtype. Future episignature testing could provide additional evidence to further support this diagnostic assignment.

Our review of reported MKHK cases (summarized in Supplementary Figure S1) (Haghshenas et al., 2024; Banka et al., 2019; Nishi et al., 2022; Sima et al., 2022; Menke et al., 2016; Menke et al., 2018; Cogan et al., 2023; Angius et al., 2019) suggests that ∼21% of CBP mutations occur in Cys residues. Their phenotypic impact is variable and lacks a clear correlation with disease severity (Haghshenas et al., 2024; Banka et al., 2019; Nishi et al., 2022; Sima et al., 2022; Menke et al., 2016; Menke et al., 2018; Cogan et al., 2023; Angius et al., 2019), including among individuals harboring identical CBP pathogenic variants (Haghshenas et al., 2024; Sima et al., 2022), or among MKHK-TAZ2 patients (Supplementary Table 1). This variability may be influenced by underlying genetic or epigenetic modifiers affecting chromatin remodeling or gene regulation during early development, potentially explaining the divergent phenotypes observed despite shared mutational hotspots.

The TAZ2 domain is a zinc-finger motif critical for CBP interactions with several transcription factors such as TP53, MYC, E1A and STAT1. Structural modeling of the identified Cys1790Arg variant indicates that it may affect the tertiary structure of TAZ2 when bound to STAT1 but not when bound to adenovirus E1A (Figure 3). Conversely, Cys1819Tyr is predicted to disrupt hydrogen bonding and to alter residue exposure in the context of E1A but not STAT1 interaction (Figure 3). These *in silico* findings raise the possibility that substitutions of different Cys residues within TAZ2 may differentially affect the interactions of CBP with specific transcription factors, likely leading to differences in the spectrum or severity of phenotypic outcomes.

Whereas this structural modeling provides valuable insights, experimental studies will be required to confirm the predicted effects of TAZ2 variants on transcription factor interactions and transcriptional activity. Interestingly, elevated levels of STAT1 activity in the developing brain of *Smc3* ^*+/−*^ mice bearing reduced levels of Cohesin have been linked to an abnormal neuronal and behavioral phenotype reminiscent of CdL syndrome (Fujita et al., 2017). STAT1 has also been reported to mediate retardation in bone development downstream of a mutated, constitutively active FGFR3 in thanatophoric dysplasia type II dwarfism (Su et al., 1997). In this regard, it would be of interest to examine whether intellectual disability and the growth retardation phenotype associated with Cys1790Arg and perhaps other CBP TAZ2 variants (Supplementary Table 1) may involve increased transcriptional activity of STAT1.

Conversely, TAZ2 variants such as Cys1819Tyr which is predicted to affect interactions of CBP with E1A, may preferentially impact viral response pathways (Ferrari et al., 2014; Zemke and Berk, 2017) or other cellular processes regulated by E1A-like transcription factors. E1A has been reported to have a dual effect on histone acetyltransferase (HAT) activity of CBP/p300, with studies indicating both inhibitory (Chakravarti et al., 1999; Hamamori et al., 1999) and activating (Ait-Si-Ali et al., 1998) roles. E1A has also been reported to compete with NFAT for binding to p300/CBP, thereby inhibiting NFAT-dependent gene expression during the immune response (Garcia-Rodriguez and Rao, 1998). Interestingly, patients bearing a Cys1819Tyr or Cys1819Phe CBP variant display recurrent infections (Haghshenas et al., 2024), whereas the Cys1790Arg MKHK patient described in this report does not. Experimental studies are warranted to validate these correlations and their impact on the divergent phenotypes associated with CBP TAZ2 variants.

## 5 Conclusion

The novel pathogenic *CREBBP* NM_004380.3:c.5368T>C p.(Cys1790Arg) variant identified in this study adds to the limited catalog of *CREBBP/EP300* variants and enriches the knowledge of the genetic basis of MKHK. We propose that different TAZ2 domain variants may affect interactions with different transcription factors, which could explain why individuals carrying mutations within the same CBP domain exhibit differences in developmental trajectories and clinical manifestations.

## Data Availability

The datasets presented in this study can be found in online repositories. The names of the repository/repositories and accession number(s) can be found below: https://www.ncbi.nlm.nih.gov/clinvar/, SCV005880094.

## Supplementary material

SUPPLEMENTARY FIGURE S1 Schematic representation of the various CBP domains (NP_004371.2) (not to scale) and summary of the pathogenic / likely pathogenic variants identified within the ZZ, TAZ2 and ID4 regions of CBP that have been linked to pathological manifestations of MKHK syndrome. The CBP variant identified in the present study is highlighted in red.

SUPPLEMENTARY TABLE S1 Summary of main clinical and anthropometric features of previously reported MKHK-TAZ2 patients vs. the patient described in this case report.
