# Supplementary material for: Identification of a novel, pathogenic CREBBP variant in a patient with Menke-Hennekam syndrome: a Case Report
Source: Front Genet. 2025 Aug 11;16:1585453. doi: 10.3389/fgene.2025.1585453 (PMC12375898; doi:10.3389/fgene.2025.1585453)
Supplement: Supplementary file 1 [file Table1.docx]

| Clinical Characteristic | Reported MKHK-TAZ2 Cases ( Haghshenas et al., 2024; Nishi et al., 2022; Sima et al, 2022) | This report |
| --- | --- | --- |
| Intellectual Disability | 8/13(62%) severe | Yes, severe |
| Behavioral Problems incl. ASD | 8/12 (67%) | No |
| Strabismus | 9/14 (64%) | Yes |
| Recurrent Infections | 7/13 (54%) | No |
| Feeding Problems | 13/14 (93%) | Yes |
| Gastroesophageal Reflux | 7/10 (70%) | No |
| Constipation | 6/11 (55%) | Yes, resolving |
| Dental Anomalies | 5/12 (42%) | Yes (hypodontia) |
| Cryptorchidism | 3/8 of males (38%) | No (normal) |
| Muscle Hypertonia | 4/13 (31%) | Yes |
| Contractures | 3/12 (25%) | No |
| Anomalies of Extremities | 7/14 (50%) | Yes (hypoplastic 5th toenail, palmar skin) |
| Thick Eyebrows | 7/13 (54%) | Mild synophrys, sparse hair |
| Broad Nasal Tip | 8/14 (57%) | Yes |
| Sex (Male/Female) | 8/6 (57%/43%) | Male |
| Gestational Age (weeks) | 38.6 (range 34–41) | 36 weeks |
| Prematurity (<37w) | 2/14 (14%) | Yes |
| Birth Weight (mean ± SDS) | -1.3 ± 1.3 SDS | 2.1 kg |
| Walking Age | 2.9 ± 1.7 years | Walks with support at 4 years |
| Speech Development | 2.3 ± 0.6 years | No speech, makes noises at 4 years |
| Autism Diagnosis | 4/12 (33%) | No |
| Visual Impairment | 3/14-9/14 (21%–64%) | Unremarkable but difficult to assess |
| Hearing Impairment (ENT) | 5/11 (45%) | No (otitis media present) |
| Cardiovascular Anomaly | 2/12 (17%) | No, normal ECG and ECHO |
| Renal Anomaly | 2/12 (17%) | No |
| Scoliosis | 1/12 (8%) | No |
| Hip Dysplasia | 2/12 (17%) | No |

*Anna Μaria Anastasiou et al. : Identification of a novel, pathogenic CREBBP variant in a patient with Menke-Hennekam Syndrome: a case report*

***Supplementary Table 1***
