# Supplementary figures and images for: Identification of a novel, pathogenic CREBBP variant in a patient with Menke-Hennekam syndrome: a Case Report
Source: Front Genet. 2025 Aug 11;16:1585453. doi: 10.3389/fgene.2025.1585453 (PMC12375898; doi:10.3389/fgene.2025.1585453)

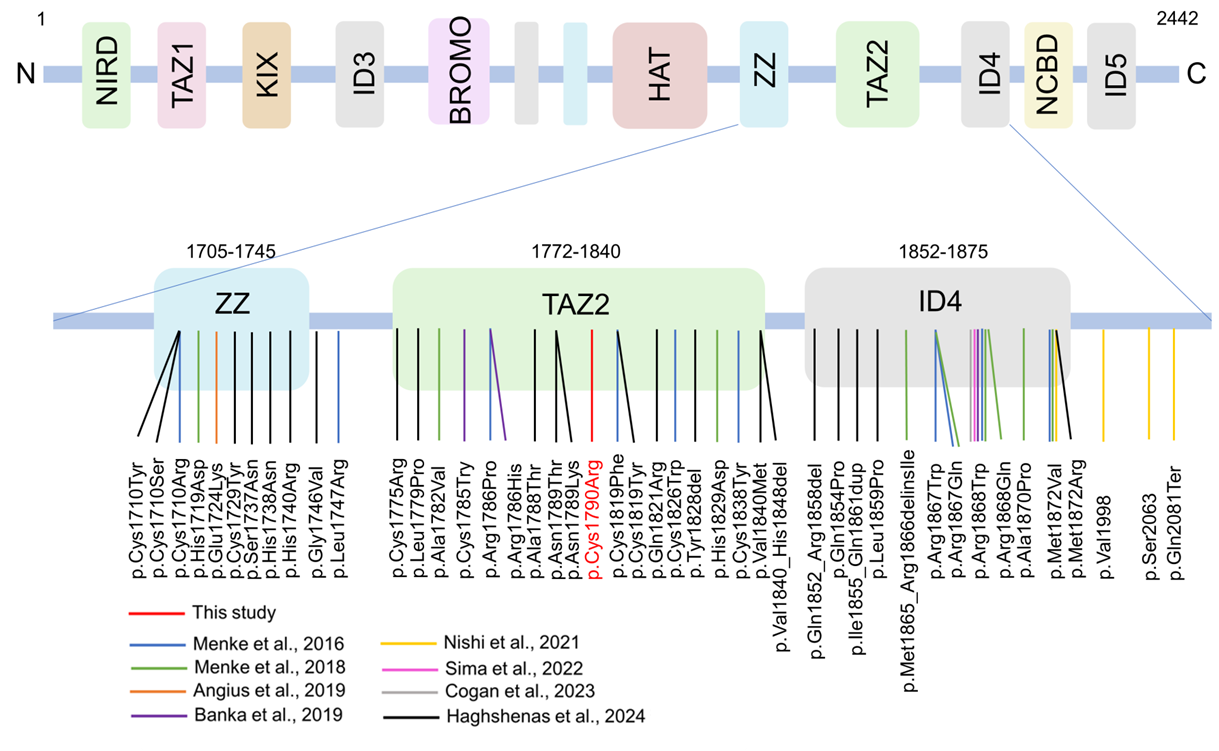

Supplement: Supplementary file 2 [file Image1.png]
